# Immune Mechanisms in Myelodysplastic Syndrome

**DOI:** 10.3390/ijms17060944

**Published:** 2016-06-15

**Authors:** Andreas Glenthøj, Andreas Due Ørskov, Jakob Werner Hansen, Sine Reker Hadrup, Casey O’Connell, Kirsten Grønbæk

**Affiliations:** 1 Epi-/Genome Laboratory, Department of Hematology, Rigshospitalet, Copenhagen University Hospital, Copenhagen 2100, Denmark; a.oerskov@gmail.com (A.D.Ø.); jakobwerner@gmail.com (J.W.H.); Kirsten.Groenbaek@regionh.dk (K.G.); 2 Section for Immunology and Vaccinology, National Veterinary Institute, Technical University of Denmark, Frederiksberg 1870, Denmark; sirha@vet.dtu.dk; 3 Jane Anne Nohl Division of Hematology, USC Norris Comprehensive Cancer Center, Los Angeles, CA 90033, USA; OConnell_C@med.usc.edu; 4 Stand up to Cancer Epigenetics Dream Team, Van Andel Research Institute, Grand Rapids, MI 49503, USA

**Keywords:** myelodysplastic syndrome, autoimmunity, adaptive immunity, innate immunity, Pancytopenia

## Abstract

Myelodysplastic syndrome (MDS) is a spectrum of diseases, characterized by debilitating cytopenias and a propensity of developing acute myeloid leukemia. Comprehensive sequencing efforts have revealed a range of mutations characteristic, but not specific, of MDS. Epidemiologically, autoimmune diseases are common in patients with MDS, fueling hypotheses of common etiological mechanisms. Both innate and adaptive immune pathways are overly active in the hematopoietic niche of MDS. Although supportive care, growth factors, and hypomethylating agents are the mainstay of MDS treatment, some patients—especially younger low-risk patients with HLA-DR15 tissue type—demonstrate impressive response rates after immunosuppressive therapy. This is in contrast to higher-risk MDS patients, where several immune activating treatments, such as immune checkpoint inhibitors, are in the pipeline. Thus, the dual role of immune mechanisms in MDS is challenging, and rigorous translational studies are needed to establish the value of immune manipulation as a treatment of MDS.

## 1. Introduction

Myelodysplastic syndrome (MDS) encompasses a range of diseases, characterized by inefficient hematopoiesis leading to debilitating cytopenias [1]. The spectrum of MDS ranges from indolent disease without the need for blood product transfusion to borderline acute myeloid leukemia (AML), and may arise *de novo*, secondary to other myeloid disorders, or after exposure to cytotoxic therapy (therapy-related MDS). Even in the novel 2016 World Health Organization classification of MDS, diagnostics are almost purely based on cytological and histological examination of peripheral blood and bone marrow, offering limited characterization of the biology of the disease. The only exception are del(5q) and *SF3B1*, which contribute to the diagnostic criteria of MDS, with isolated del(5q) and MDS with ring sideroblasts, respectively [2]. Recent years have uncovered a large spectrum of point mutations in MDS, the majority being in splicing factors and epigenetic regulators [3,4,5]. Such mutations reflect clinical outcomes [6], relate to clinical phenotypes [7], and will likely be incorporated into future prognostic scoring systems. However, no somatic mutations have proven pathognomonic of MDS, as these mutations can be detected in patients with other myeloid malignancies and in people without dysplastic bone marrow [8,9]. Individuals with these mutations may be healthy and have normal blood counts or idiopathic cytopenia of undetermined significance (ICUS). A fraction of these will develop MDS over time [9], but the factors determining progression have yet to be identified.

A paramount feature of low-risk MDS is increased apoptosis of hematopoietic precursors [10,11]. Both genetic and epigenetic alterations, as well as immune mechanisms, may contribute to this phenomenon [12]. Immune dysregulation has been intensively studied and has repeatedly been reported as the cause of apoptosis in MDS [12]. Furthermore, many patients seem to benefit from immunosuppressive therapy [13]. Epidemiological studies of large cohorts have established a link between autoimmune disease and MDS [14,15,16]. In this review, we explore the link between immune dysregulation and MDS, and its role in current and potential future treatment of the disease. 

## 2. Association with Autoimmune Disease

Myelodysplasia has long been associated with autoimmune diseases on an anecdotal basis. More recently, large cohort studies have confirmed such a link [14,15,16,17,18]. Database studies have shown an increased odds ratio (OR) of between 1.5 to 3.5 for developing MDS in patients with prior autoimmune disease [14,16,18,19]. However, these epidemiological studies lack data on treatment given for the autoimmune disease. Some patient groups have probably received immunosuppressive drugs, such as methotrexate, azathioprine, or cyclophosphamide, which may induce therapy-related MDS [20,21,22]. Looking at the autoimmune diseases individually in a Swedish study, some diseases had a particularly high OR of 23.9 for idiopathic thrombocytopenic purpura (ITP), 7.9 for myasthenia gravis, and 5.4 for giant cell arteritis [16]. One study found hypothyroidism in 12% of patients (<4% in the general population), but it was not verified if hypothyroidism was of autoimmune origin (Hashimoto’s thyroiditis) [17]. Another study from the General Practice Research Database in the United Kingdom failed to replicate this association, but found a strong relation to systemic lupus erythematosus and inflammatory bowel disease [18]. Psoriasis and rheumatoid arthritis are other diseases overrepresented in MDS [17]. One could suspect some surveillance bias (*i.e.*, surveillance of autoimmune disease leads to faster MDS diagnosis), but adjustment for number of visits to the General Practitioner did not alter the increased risk found in the United Kingdom study [18]. Taken together, these studies suggest that autoimmunity could underlie both hematological and systemic autoimmune morbidity. On the other hand, idiopathic cytopenia of undetermined significance (ICUS) that may precede MDS [8,9] may also be associated with immune dysregulation or even autoimmune manifestations. One might also speculate that patients with presumed idiopathic thrombocytopenic purpura (ITP) and subsequent MDS may have had clonal disease all along. Studies from our own lab show that some MDS patients have a preceding clonal thrombocytopenia for up to eight years before developing MDS, and a subset of patients with ITP have detectable mutations in epigenetic regulators without progressing to MDS [23]. It is conceivable that progression from ITP to MDS can be driven by a compromised immune system. 

In general, patients with MDS and concurrent autoimmune disease fail to demonstrate consistent characteristic demographic, clinical, or paraclinical features [15,17]. Prognostically, one study found prior autoimmune disease to be a beneficial predictor of longer survival and less progression to acute myeloid leukemia (AML) [17], although another smaller study did not detect any significant difference [15].

## 3. Immune Dysregulation in MDS

### 3.1. T-Cell Mediated Bone Marrow Suppression

**Figure 1:**
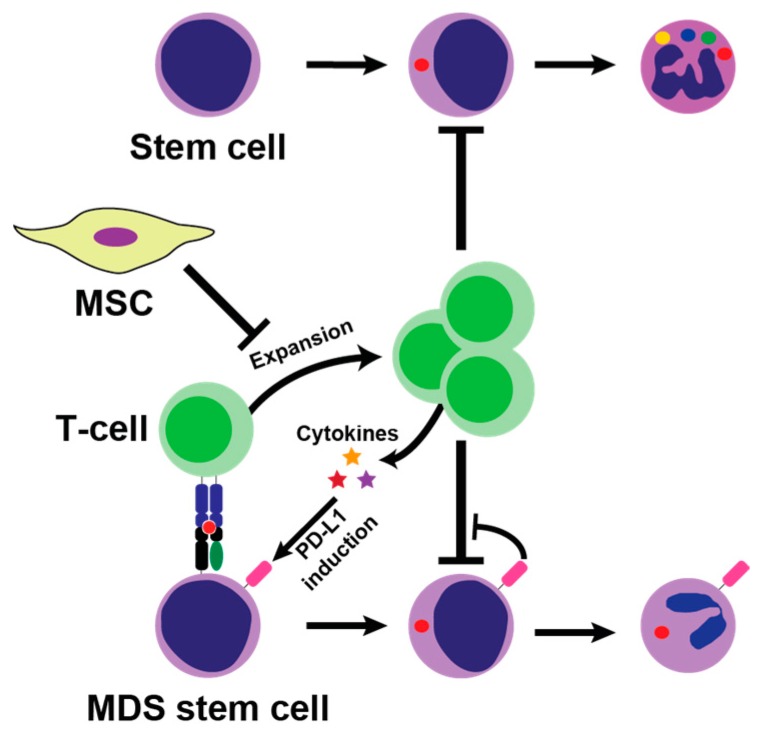
Potential mechanism of bone marrow suppression by T-cells. CD8+ T-cells are activated by major histocompatibility complex (MHC)-class I complex on malignant MDS stem cells, which leads to T-cell expansion which in turn suppress hematopoiesis. T-cells secrete proinflammatory cytokines such as tumor necrosis factor (TNF)-α and interferon gamma (IFN)-γ into the bone marrow microenvironment, which may both hinder hematopoiesis and induce PD-L1 on tumor cells hereby promoting escape from tumor surveillance. Normally, mesenchymal stromal cells (MSC) suppress T-cells activation in the bone marrow by paracrine and cell-to-cell interaction, but these mechanisms may be flawed in myelodysplastic syndrome (MDS).

Apoptosis is the hallmark of low-risk MDS [11] and may be attributed to dysfunctional T-cell response and innate immune activation [12,24]. In MDS, naïve T-cells (CD3+) exhibit shorter telomere length and have significantly less proliferative potential [25]. More than 90% of MDS-patients harbor oligoclonal T-cells [26], and most of these are derived from the malignant MDS-clone [27]. *In vitro* studies have demonstrated that autologous T-cells inhibit growth of both malignant and non-malignant hematopoiesis [28,29,30]. This may be mediated through CD8+ T-cells targeting MHC-class I molecules on hematopoietic precursors (Figure 1). Although clonal repertoires have been described through flow cytometry and PCR of the T-cell receptor (TCR), these methods cannot themselves identify the epitopes targeted on hematopoietic stem cells in MDS. A recent study was able to identify Wilms tumor 1 protein as a potential epitope for autologous T-cells in trisomy 8 MDS [31]. Another study demonstrated a T-cell reactivity against cancer-testes antigens in MDS, a response that was potentiated by treatment with the hypomethylating agent (HMA) azacitidine [32]. This autologous T-cell response is a part of tumor surveillance. Many of the IST regimes tested in MDS affect T-cell function, which could hinder tumor surveillance and induce disease progression. Contrary to this notion, clinical trials actually demonstrated similar or less risk of progression to AML after immunosuppressive therapy [33]. An explanation may be that MDS tumor cells already have escaped tumor surveillance through a variety of mechanisms ranging from dysfunctional T-cells and cytokine expression to altered stroma in the hematopoietic niche [34,35,36,37]. Recently, suppressors of activated T-lymphocytes, such as programmed death-1 (PD-1), its ligand programmed death ligand 1 (PD-L1), and T lymphocyte-associated antigen 4 (CTLA4), have attracted a great deal of attention in oncology [38]. In MDS, these seem to play an active role in escape of tumor surveillance and resistance to therapy [34,39,40]. 

T-cell mediated suppression of hematopoietic stem cells has been recognized as an attribute of aplastic anemia [41]; distinction of this entity from hypoplastic MDS can be difficult. Recent advances in the mutational mapping have demonstrated that almost half of the patients with aplastic anemia harbor mutations characteristic of MDS, indicating that at least some of these patients may indeed have MDS associated with T-cell activation [42]. 

### 3.2. Cytokines

The expression of at least thirty cytokines has been found to be skewed in patients with MDS [12,43,44] and some patterns relate to the clinical subtype and outcome [12]. These cytokines may be expressed by MDS tumor cells, stromal cells or they may reflect systemic inflammatory activity. Tumor necrosis factor alpha (TNF-α) is well-studied in MDS; it is overexpressed in cultured cells from patients with MDS [45] and elevated in bone marrow and peripheral blood plasma [44,46,47]. The level of TNF-α appears to be inversely related to hemoglobin and survival [47]. Stromal bone marrow cells are one source of TNF-α [43], but systemic inflammatory activity may also be a significant source. Furthermore, interferon-γ (IFN-γ) is secreted locally by activated T-cells [48] as well as by stromal macrophages [43]. *In vitro*, TNF-α and INF-γ greatly enhance the expression of the Fas receptor (FasR) on hematopoietic precursors [49], which renders them susceptible to Fas ligand (FasL) mediated apoptosis. Tumor necrosis factor–related apoptosis-inducing ligand (TRAIL) and its agonistic receptors show significant overactivity in MDS leading to apoptosis of precursors with some preference for the malignant clone [50]. 

### 3.3. Innate Immunity Activity

Innate immunity is composed of humoral and cellular immune mechanisms, which mostly rely on generic recognition of microbial markers through pattern recognition receptors. In mammals, this recognition most often relies on toll-like receptors (TLRs) [51]. The TLR signaling pathway results in activation of the nuclear factor k-light-chain-enhancer of activated B cells (NF-kB) and mitogen-activated protein kinase (MAPK) pathways, which in turn induce transcription of pro-inflammatory cytokines [51]. In many cases of MDS, TLR signaling pathways are severely overactive due to overexpression of activators such as MYD88, TIRAP, IRAK1/4, TRAF and downregulation of inhibitory factors, such as miR145 and miR146a [12,52]. MicroRNAs (miRNAs) are small nucleotides used for post-translational regulation of Mrna [53] and their role in regulating hematopoiesis is well described [54,55]. MiR145 and miR146a are both deleted in 5q- MDS [52]. MiR145 and targets TIRAP, whereas miRNA146b works downstream of that by inhibiting TRAF6 [52]. 

### 3.4. Mesenchymal Stromal Cells (MSC)

Hematopoiesis occur in the hematopoietic niche in which stems cells are nurtured by supporting MSC [56]. MSC are primitive and self-renewing cells capable of differentiating into mesodermal cell types such as adipocytes, chondrocytes, and osteocytes [56]. Increasingly, MSC are recognized as pivotal in maintaining normal hematopoiesis as well as in fueling the pathogenesis of the malignant MDS clone [57]. This is illustrated by the inability of human MDS stem cells to engraft in murine xenograft models. Engraftment is substantially improved by injecting MSC along with hematopoietic MDS stem cells into the bone marrow of xenograft mice [37]. Additionally, altered gene and miRNA expression profiles of have been reported in MSC of patients with MDS [58]. One gene differentially expressed in MSC of MDS patients, *Dicer1*, has been selectively deleted in MSC in a murine model, and this alteration alone was shown to induce MDS and AML [59]. Notably, in the context of this review, MSC exert substantial immunosuppressive activity by paracrine and cell-to-cell interaction [60]. T-cells are arrested in G_1_-phase and their cytokine secretion is diminished [61,62,63]. One effector of this T-cell inhibition is indoleamine 2,3-dioxygenase (IDO), which is secreted by MSC [64]. As described below, clinical trials are investigating the potential of IDO in treatment of several malignancies including MDS. MSC themselves secrete a broad range of other cytokines capable of modulating numerous leukocyte subsets [64]. Importantly, this immunomodulation differs between low- and high-risk MDS patients with MSC from high-risk patients inducing more apoptosis and immunosuppression while providing less hematopoietic support [60]. 

## 4. Immune Manipulation as Treatment of MDS

As both innate and adaptive immune reactivity seem to participate in the development of low-risk MDS, immunosuppressive therapy would be a rational treatment in this diseases entity. However, in high-grade MDS, escape of immune surveillance by the adaptive immune system may be crucial for MDS blast survival [12,39,65], thus providing yet another target for therapy. Several trials have examined the potential effect of immune manipulation in low- and high-risk MDS, which will be briefly reviewed in the following. 

### 4.1. Which Low-Risk MDS Patients Are Subceptible to Immunosuppressive Therapy

As low-risk MDS represents a range of disease entities, the susceptibility to immunosuppressive therapy differs widely. Age is strongly and inversely related to immunosuppressive therapy response [33]. Other factors associated with response to immunosuppressive therapy are HLA-DR15 tissue type, low international prognostic scoring system (IPSS) score, hypocellular bone marrow, and trisomy 8 [31,33,66,67]. Presence of a PNH-clone would intuitively indicate autoimmunity and thus prediction of response to immunosuppressive therapy, but this only seems the case in aplastic anemia [42] and not in MDS [33]. A recent phase II trial in patients with MDS has provided a multivariate response model incorporating younger age, short disease duration, high percent CD8+ terminal memory cells, and high percent CD4+ Ki67+ as markers of response to anti-thymocyte globulin (ATG) [68]. 

### 4.2. Cyclosporine, Anti-Thymocyte Globulin, and Mycophenolate Mofetil 

Cyclosporine specifically targets T-cell activation through binding to the cytosolic protein cyclophilin, as well as blockage of the JNK and p38 pathways [69]. Early small trials of cyclosporine monotherapy in low-risk MDS provided impressive hematological response rates of 33%–82% for anemia, whereas thrombocytopenia and neutropenia proved more treatment-resistant [70,71,72]. Anti-Thymocyte Globulin (ATG) binds to lymphocytes [73] and briefly depletes T-lymphocytes in circulation and lymph nodes. Furthermore, regulatory T-cells are upregulated and antigen presentation inhibited after ATG [74,75]. The overall result is diminished adaptive immunity [72]. Horse and rabbit ATG have been used for more than 30 years to treat aplastic anemia [76] with horse ATG as the most effective option in this disease [77]. Although considered a separate entity, hypoplastic MDS shares many features with aplastic anemia [66], and recent sequencing studies have revealed considerable overlap in somatic mutations among the two disorders [42]. This prompted studies with ATG for hypoplastic MDS and then for normo- and hyperplastic MDS [30,78]. Response rates vary between 0%–100% and greatly depend on the selection of patients [72,79]. Combination therapy of cyclosporine and ATG has not been proven superior to monotherapy, although some patients may be dependent on cyclosporine maintenance after ATG [79,80]. Mycophenolate Mofetil (MMF) is an immunosuppressant prodrug. It is metabolized in the liver to mycophenolic acid, which hinders purine synthesis in lymphocytes [81]. MMF is approved for use after organ transplantations. One small study has investigated its use in low-risk MDS patients, most of whom had failed EPO treatment [82]. Treatment was well tolerated and did not require hospitalization. Five of ten patients responded to treatment with three demonstrating major responses. 

### 4.3. Alemtuzumab

Alemtuzumab is a monoclonal antibody directed against CD52, which is primarily located on the surface of mature lymphocytes. Treatment with alemtuzumab depletes lymphocytes thus crippling adaptive immunity. In MDS, alemtuzumab has shown impressive response rates of 72% in IPSS intermediate-1 and intermediate-2 patients deemed likely to respond to immunosuppressive therapy [83]. 

### 4.4. Novel Immune Pathway Inhibitors

Improved understanding of immune mechanisms implicated in MDS offers new targets for treatment. The overactivity of TLR pathways in MDS provides a rationale for using inhibitory drugs. Many compounds have been tested preclinically. *In vitro* data suggest impressive single agent activity of p38 MAPK inhibitors [84], prompting a phase I trial of the p38 MAPK inhibitor ARRY614 (NCT01496495/NCT00113893). The TLR2 inhibitor OPN-305 has passed a phase I trial in healthy subjects and is currently being tested in a phase I/II trial in patients with MDS (NCT02363491). 

### 4.5. Epigenetic Modulation and Induction of Adaptive Immunity in Higher Risk MDS

While it is well established that accumulation of genetic and epigenetic abnormalities in the malignant blasts may aggravate MDS, it is becoming increasingly evident that evasion of adaptive immune surveillance is an important feature in higher risk MDS. One of the main mechanisms might be the adaptation of immune checkpoint molecules by the malignant cells, thereby suppressing the specific immunological anti-tumor responses [34,39]. Accordingly, a rational strategy may be to block immune checkpoints in higher-risk MDS [85].

Currently, two clinical trials are investigating the use of programmed death ligand 1 (PD-L1) inhibitors in MDS. Pembrolizumab, a PD-L1 inhibitor approved for use in melanoma, is undergoing a phase 1 trial as a single agent against MDS and lymphomas (NCT02508870). Another PD-L1 blocking antibody, Atezolizumab, is being tested with and without the HMA azacitidine in an ongoing phase 1 trial (NCT02508870). 

Cytotoxic T-lymphocyte-associated protein 4 (CTLA-4) is expressed on helper T-cells and provides an inhibitory signal to other T-lymphocytes thus acting as a checkpoint [86]. The CTLA-4 inhibitor Ipilimumab, approved in melanoma, is in phase 1 trial in relapsed/refractory MDS before or after stem cell transplantation (NCT01757639, NCT01822509).

The tryptophan degrading enzyme IDO is expressed by a range of tumor cells [87]. This exhausts L-Trp from the tumor microenvironment, which effectively dampens T-cell proliferation and activity [88]. Results from a recent phase II trial of the IDO1 inhibitor INCB024360 demonstrated stable disease in 12 of 15 patients during the follow-up period, while the remaining three patients progressed (NCT01822691). 

The HMAs that are clinically approved for the treatment of higher-risk MDS, have the potential to modulate and induce immune responses against the malignant cells in different ways [89,90,91,92]. Recently, it was shown that the mechanism of the increased immune signaling could be demethylation and upregulation of endogenous retroviruses (ERVs) [93,94]. Upregulated ERVs may form double stranded RNA that activate the viral defense pathway and contribute to decreased proliferation and apoptosis of the target cells; a phenomenon called viral mimicry [95]. This mechanism may play an important role in the anti-tumor activity of the HMAs and may sensitize malignant MDS cells to immune checkpoint blockade or other immunotherapies [96]. Additionally, upregulation of tumor associated antigens such as cancer-testis antigens, has been shown to promote T cell reactivity following HMA therapy [32,97]. Therefore, it will be of high interest to evaluate the efficacy of the combination treatment of HMAs and immune checkpoint inhibition in higher-risk MDS patients. 

## 5. Discussion

The diagnosis of MDS still mostly relies on careful examination of blood and bone marrow morphology. Karyotyping is obligatory as cytogenetic status is used in current prognostic scoring systems [98,99]. Targeted gene screening panels are becoming commercially available and will assist in classification in the next World Health Organization classification guideline as well as in future prognostic scores. Although autoimmunity has been recognized in low-risk MDS for almost two decades [71,78], the mechanisms underlying immune activation in MDS and their therapeutic implications are still sparsely understood. Initial studies have focused on adaptive immunity inducing apoptosis in hematopoietic precursors in low-risk MDS [100]. Despite an extensive search, only cancer-testes antigens and WT1 antigen have been recognized as T-cell epitopes in MDS [31,32]. Therapies, such as ATG, cyclosporine, and alemtuzumab, work primarily by suppressing adaptive immunity and have shown substantial therapeutic potential for selected patients with low-risk MDS [83]. Despite impressive response rates in selected subgroups, the potential of immunosuppressive therapy is still not widely appreciated clinically. In part, this may be explained by the lack of effective tools to reliably identify patients with MDS that are likely to respond to immunosuppressive therapy. Currently, one would expect a younger patient with short duration of disease, HLA-DR15 tissue type, and low IPSS-R score to respond to immunosuppressive therapy [68,83]. Precision may be improved by adding flow cytometric characterization of T-cell populations [68]. Thus far, effective immunosuppressive therapy in MDS has mostly been built on trial and error with known methodologies working in other autoimmune diseases. Identifying novel markers and targets of autoimmunity in MDS will require deeper understanding of its underlying biological mechanisms. 

Recently, adaptive immunity has attracted much attention in high-risk MDS. As with melanoma [38], harnessing tumor surveillance by pharmacologically releasing cytotoxic lymphocytes from their bonds may prove instrumental in providing lasting responses in higher risk MDS patients. However, given the role of autoimmunity in low-risk MDS, careful monitoring of adverse effects of immune check point inhibitors in higher risk MDS will be crucial, as these drugs are associated with increased risk of autoimmunity. Increasingly, evidence of innate immunity dysregulation in the hematopoietic niche of MDS is also mounting. This is paving the way for novel inhibitors of innate immune pathways and may widen the use of immune manipulation in MDS. Such targets will hopefully provide effective and less toxic treatment options for an often fragile patient population.

## Abbreviations

AML	Acute myeloid leukemia
ATG	Anti-thymocyte globulin
ERVs	Endogenous retroviruses
FasL	Fas ligand
FasR	Fas receptor
HMA	Hypomethylating agent ICUS Idiopathic cytopenia of undetermined significance
IDO	Indoleamine 2,3-dioxygenase
IFN-γ	Interferon-γ IPSS International Prognostic Scoring System
IPSS-R	Revised International Prognostic Scoring System
ITP	Idiopathic thrombocytopenic purpura
MAPK	Mitogen-activated protein kinase
MDS	Myelodysplastic syndrome MHC Major histocompatibility complex MMF Mycophenolate Mofetil
MSC	Mesenchymal stromal cells
NF-κB	Nuclear factor k-light-chain-enhancer of activated B cells
NSAIDs	Non-steroidal anti-inflammatory drugs
RA	Refactory anemia
RCMD	Refractory anemia with multilineage dysplasia
TCR	T-cell receptor
TNF-α	Tumor necrosis factor α
TRAIL	Tumor necrosis factor–related apoptosis-inducing ligand
